# Supplementary material for: A Randomized Clinical Trial Investigating an Integrated Nursing Educational Program to Mitigate Chemotherapy-Induced Nausea and Vomiting in Cancer Patients: The NIV-EC Trial
Source: Cancers (Basel). 2023 Oct 27;15(21):5174. doi: 10.3390/cancers15215174 (PMC10649710; doi:10.3390/cancers15215174)
Supplement: Supplementary file 1 [file cancers-15-05174-s001.zip › cancers-2596226-supplementary.pdf]

Table S1. Self-reported nausea at second infusion (Functional Life Index-Emesis questionnaire).

	**Study arm (Type of information)**				**Difference % (95% CI)**	**Pearson χ^2 test**
	**Oral + written**		**Only oral**			
	**n**	**(%)**	**n**	**(%)**		
Did you experience nausea in the past five days?						
None/Not at all	143	(86.1)	123	(77.9)	8.2(-0.1 to 16.6)	0.052
A little/Moderately	18	(10.8)	18	(11.4)	-1.4 (-7.4 to 6.3)	0.875
A lot/A great deal	5	(3.0)	17	(10.8)	**-7.8 (-13.2 to -2.3)**	**0.006**
Did the nausea affect your ability to pursue leisure activities?						
None/Not at all	142	(85.5)	130	(82.3)	3.3 (-4.7 to 11.3)	0.424
A little/Moderately	20	(12.1)	15	(9.5)	2.6 (-4.2 to 9.3)	0.459
A lot/A great deal	4	(2.4)	13	(8.2)	**-5.8 (-10.7 to -0.9)**	**0.019**
Did the nausea affect your ability to prepare food or do small houseworks?						
None/Not at all	137	(82.5)	124	(78.5)	4.0 (-4.6 to 12.7)	0.357
A little/Moderately	23	(13.9)	22	(13.9)	0.0 (-7.6 to 7.5)	0.986
A lot/A great deal	6	(3.6)	12	(7.6)	-4.0 (-9.0 to 1.0)	0.118
How much has nausea affected your urge to eat?						
None/Not at all	124	(74.7)	118	(74.7)	0.0 (-9.5 to 9.5)	0.998
A little/Moderately	28	(16.9)	24	(15.2)	1.7 (-6.3 to 9.7)	0.681
A lot/A great deal	14	(8.4)	16	(10.1)	-1.7 (-8.0 to 4.6)	0.599
How much did nausea affect your ability to enjoy a drink?						
None/Not at all	122	(73.5)	115	(72.8)	0.7 (-8.9 to 10.4)	0.886
A little/Moderately	30	(18.1)	24	(15.2)	2.9 (-5.2 to 11.0)	0.487
A lot/A great deal	14	(8.4)	19	(12.0)	-3.6 (-10.2 to 3.0)	0.285
How much did the nausea affect your desire to see relatives/friends and spend time with them?						
None/Not at all	130	(78.3)	122	(77.2)	1.1 (-8.0 to 10.2)	0.812
A little/Moderately	31	(18.7)	24	(15.2)	3.5 (-4.7 to 11.6)	0.404
A lot/A great deal	5	(3.0)	12	(7.6)	-4.6 (-9.4 to 0.3)	0.064
Did the nausea affect your daily activities?						
None/Not at all	134	(80.7)	121	(76.6)	4.1 (-4.8 to 13.6)	0.363
A little/Moderately	25	(15.1)	24	(15.2)	-0.1 (-7.9 to 7.7)	0.974
A lot/A great deal	7	(4.2)	13	(8.2)	-4.0 (-9.3 to 1.3)	0.134
Do you think that the nausea has put a strain on you?						
None/Not at all	139	(83.7)	122	(77.2)	6.5 (-2.1 to 15.1)	0.138
A little/Moderately	20	(12.1)	25	(15.8)	-3.7 (-11.3 to 3.8)	0.326
A lot/A great deal	7	(4.2)	11	(7.0)	-2.8 (-7.8 to 2.3)	0.281
Do you think that the nausea has put a strain on your relatives/friend?						
None/Not at all	137	(82.5)	128	(81.0)	1.5 (-6.9 to 9.9)	0.724
A little/Moderately	21	(12.7)	18	(11.4)	1.3 (-5.8 to 8.3)	0.728
A lot/A great deal	8	(4.8)	12	(7.6)	-2.8 (-8.0 to 2.5)	0.299

CI: Confidence interval
